# A sex- and gender-based analysis of alcohol treatment intervention research involving youth: A methodological systematic review

**DOI:** 10.1371/journal.pmed.1004413

**Published:** 2024-06-03

**Authors:** A.J. Lowik, Caroline Mniszak, Michelle Pang, Kimia Ziafat, Mohammad Karamouzian, Rod Knight

**Affiliations:** 1 British Columbia Centre on Substance Use, Vancouver, Canada; 2 Institute for Gender, Race, Sexuality and Social Justice, University of British Columbia, Vancouver, Canada; 3 Department of Medicine, University of British Columbia, Vancouver, Canada; 4 Centre on Drug Policy Evaluation, Saint Michael’s Hospital, Toronto, Canada; 5 Université de Montréal, École de santé publique, Montréal, Canada; 6 Centre de recherche en santé publique (CReSP), Montréal, Canada; London School of Hygiene and Tropical Medicine, UNITED KINGDOM

## Abstract

**Background:**

While there is widespread consensus that sex- and gender-related factors are important for how interventions are designed, implemented, and evaluated, it is not currently known how alcohol treatment research accounts for sex characteristics and/or gender identities and modalities. This methodological systematic review documents and assesses how sex characteristics, gender identities, and gender modalities are operationalized in alcohol treatment intervention research involving youth.

**Methods and findings:**

We searched MEDLINE, Embase, Cochrane Central Registry of Controlled Trials, PsycINFO, CINAHL, LGBT Life, Google Scholar, Web of Science, and grey literature from 2008 to 2023. We included articles that reported genders and/or sexes of participants 30 years of age and under and screened participants using AUDIT, AUDIT-C, or a structured interview using DSM-IV criteria. We limited the inclusion to studies that enrolled participants in alcohol treatment interventions and used a quantitative study design. We provide a narrative overview of the findings.

Of 8,019 studies screened for inclusion, 86 articles were included in the review. None of the studies defined, measured, and reported both sex and gender variables accurately. Only 2 studies reported including trans participants. Most of the studies used gender or sex measures as a covariate to control for the effects of sex or gender on the intervention but did not discuss the rationale for or implications of this procedure.

**Conclusions:**

Our findings identify that the majority of alcohol treatment intervention research with youth conflate sex and gender factors, including terminologically, conceptually, and methodologically. Based on these findings, we recommend future research in this area define and account for a spectrum of gender modalities, identities, and/or sex characteristics throughout the research life cycle, including during study design, data collection, data analysis, and reporting. It is also imperative that sex and gender variables are used expansively to ensure that intersex and trans youth are meaningfully integrated.

**Trial registration:**

**Registration:** PROSPERO, registration number: CRD42019119408

## Abstract 2

A.J. Lowik systematically review the consideration and reporting of sex and gender variables in studies of alcohol treatment interventions involving young people.

## Author summary

**Why was this study done?:**

- Both sex and gender are important factors for intervention design, implementation, and evaluation, including with regards to alcohol treatment interventions for young people. However, little is known about how alcohol treatment research accounts for sex and gender factors.

**What did the researchers do and find?:**

- We systematically searched the peer-reviewed literature to identify alcohol treatment intervention studies that reported genders and/or sexes of participants 30 years of age or younger.
- Of the 86 articles included in our review, we found that none of them defined, measured, and reported both sex and gender variables accurately. Approximately 37% (*n* = 32) of the studies defined, measured, and reported either sex or gender accurately. Only 2 studies reported including trans participants.
- Most of the studies (*n* = 54) used sex or gender measures to control for their effects on the intervention but did not discuss the implications of this procedure.

**What do these findings mean?:**

- Our findings identify how the vast majority of alcohol treatment intervention research with youth conflates sex and gender factors, including terminologically, conceptually, and methodologically.
- To advance sex and gender science in alcohol treatment intervention research, it is essential that researchers clearly articulate why they are choosing to include measures related to sex, gender or both, and to advance study designs and procedures that can account for sex and gender.
- It is also imperative that sex and gender variables are used in a way that ensures that intersex and trans people are meaningfully integrated so that both research and intervention can address their alcohol-related needs.

## Introduction

While there is widespread consensus that sex- and gender-based factors are important for how interventions are designed, implemented, and evaluated [1,2], it is not currently known how alcohol treatment intervention research accounts for sex characteristics and/or gender identities and modalities. This knowledge gap is particularly salient for youth who experience harms from alcohol more intensely, given that experiences with regular or high-risk binge drinking during early phases of the life course (e.g., adolescence, young adulthood) increases the risks for alcohol-related harms to occur during subsequent phases of the life course [3–5]. Critically, both acute and chronic alcohol-related outcomes are impacted by a variety of sex- and gender-related factors. For example, epidemiological data across a variety of settings identifies how adolescent boys tend to initiate alcohol use earlier than adolescent girls, and that young adult men tend to drink in excess more regularly than young adult women [6] (note: we attempted to specify whether the literature cited is *trans*-inclusive or *cis*-specific; however, in most cases, this was not possible as the literature reviewed does not specify whether the study population included trans people). Research also documents how lifetime risks of health harms increases more steeply for women than for men when alcohol consumption occurs above low levels and when initiated from an early age, including during adolescence and young adulthood [1,5]. More recently, there has been a narrowing of the differences in chronic health outcomes associated with long-term drinking patterns between men and women, despite a long-standing body of evidence indicating that these outcomes are more persistently reported among men compared to women. This trend is observed in some settings, including the United States [7]. There is also a small but growing evidence base documenting how trans people experience higher rates of alcohol use when compared to their cisgender counterparts [7–10], though youth-specific data remains limited.

Clinical research has documented how sex-related factors are important in understanding how alcohol is absorbed, metabolized, and eliminated in bodies that are assigned male and female at or before birth, including via human physiology, anatomy, hormones, enzymes, genetics, and neurobiology [2,5]. Overall, this body of research documents that above low levels of alcohol consumption, female-assigned bodies are more likely to experience organ and other bodily damage and disease [1,5]. Social scientific, behavioral and epidemiological research also documents how gender-related factors impact population-level alcohol use patterns and outcomes, including with regards to gender roles and norms, gender relations, gender identities, and institutionalized gender [1]. For example, sociocultural and gender norms contribute to patterns in which men, on average, tend to drink in excess more than women and are also more likely to engage in high-risk behavior when intoxicated [11]. Indeed, the higher prevalence among young men of alcohol-impaired driving collisions [12] and other alcohol-related medical emergencies and health problems—including death [11]—are largely attributed to gender factors. Elevated rates of alcohol use among trans people of all genders are also attributed to social and structural factors, including exposure to minority stressors such as stigma, violence, and discrimination [8,13]. It has also been documented that, when intoxicated, cis girls and women and trans people of all genders are more vulnerable to sexual assault [14] and intimate partner violence (IPV) [11]; conversely, cisgender men and boys are more likely to be involved as perpetrators of alcohol-related violence [15].

Given that sex- and gender-based differences are critically important to alcohol-related outcomes among youth, it is important that the science informing alcohol treatment intervention development in this area attends to sex and gender concepts accurately [16,17]. For the current review, we turn our attention towards research involving alcohol treatment interventions that seek to address problematic alcohol use among youth, including psychosocial or behavioral interventions (e.g., cognitive behavioral therapy) and pharmacological treatments (e.g., antagonist treatment therapies). Behavioral therapies, such as cognitive-behavioral therapy and motivational therapy, as well as family-based approaches, have all demonstrated varying degrees of efficacy in treating alcohol use disorders among youth [18]. Although pharmaceutical therapies are not commonly used to treat alcohol use disorders among youth in most jurisdictions, research has demonstrated that these approaches can be helpful in some circumstances [18], particularly when combined with psychological and behavioral treatments [19,20]. Given that little is known about how sex- and gender-related factors are assessed and reported within the youth-focused alcohol treatment intervention evidence base, the objective of this study is to provide a methodological systematic review to document and assess how sex characteristics, gender identities, and gender modalities are operationalized in alcohol treatment intervention research involving youth, including adolescents and young adults. Our overarching research question is: How are gender and sex measured and reported in research on alcohol treatment for youth up to age 30?

## Method

We registered our study protocol on PROSPERO (registration number: CRD42019119408) and followed the Preferred Reporting Items for Systematic Reviews and Meta-Analyses (PRISMA) 2020 checklist for reporting [21]. Changes to our PROSPERO protocol are inventoried in Appendix A in S1 Appendices.

### Search strategy

We searched MEDLINE (Ovid), Embase (Ovid), Cochrane Central Registry of Controlled Trials (CENTRAL), PsycINFO (EBSCOhost), CINAHL (EBSCOhost), LGBT Life (EBSCOhost), the first 300 citations on Google Scholar [22], and Web of Science for studies involving alcohol treatment among youth. Grey literature was identified using GreyMatter, des Libris (http://deslibris.ca), OpenGrey (www.opengrey.eu), and via custom Google searches; each source was last consulted as of January 4, 2024. As part of the review process, we manually examined the reference lists of all included articles, as well as the articles that cited them, and any review papers identified during the screening stage to identify additional relevant articles. The search was restricted to articles published between January 1, 2008 and December 31, 2023, to keep the work feasible and relevant. See Appendix B in S1 Appendices or the full search details for Medline.

### Eligibility criteria

The population, interventions, comparisons, outcomes, and study designs considered for review are listed in Table 1.

**Table 1:** PICOS.

**Population**	(a) Participants had their sexes and/or genders gathered and recorded in the data^1^. (b) Participants were less than 30 years old at the time of data collection^2^. (c) Participants were screened using AUDIT^3^, AUDIT-C, or a structured interviewing using DSM-IV criteria^4^ for problematic alcohol use, and screening occurred as part of the study activities^5^.
**Intervention**	(d) Participants enrolled in a psychosocial and/or pharmacological alcohol treatment intervention as part of the study’s design.
**Comparisons**	(e) Placebo or other/no interventions.
**Outcomes**	None specified.
**Study Design**	(f) Study types considered included: quantitative randomized studies (controlled or uncontrolled) and quasi-experimental studies.

^1^This eligibility criterion was imperative, as the primary outcome of interest in this systematic review was an analysis of how sex and/or gender were measured, gathered, and reported in youth-focused alcohol intervention research.

^2^Where the mean and/or median age of the study participants was reported as less than 30 years old; or, where no mean and/or median was reported, where the age range of participants was described as including only those participants below 30 years old. This eligibility criterion was informed by our team’s prior experience with youth-focused research, by the state of public health research regarding youth and alcohol use, as well as a set of observable secular trends among individuals within this age range, including delayed transitions associated with adulthood, such as delays in leaving home and achieving financial independence [23]. We therefore consider those under 30 years old as youth.

^3^The Alcohol Use Disorders Identification Test, a 10-item screening tool developed by the World Health Organization to assess alcohol consumption, drinking behaviors and alcohol-related problems.

^4^The Diagnostic and Statistical Manual of Mental Disorders, Fourth Edition, which includes criteria for substance use disorder diagnoses.

^5^These tools were chosen as a condition of inclusion for this review due to their use as diagnostic tools in research and treatment settings [18,24,25]. We made this decision because, during our initial searches, we found great heterogeneity across the literature with regards to how various studies described their inclusion/exclusion based on alcohol use of their study samples. By way of 2 examples, one study we assessed reported including participants for treatment interventions based on violations of college or university student drinking policies while another on blood-alcohol levels indicating intoxication on a single occasion. Given that the AUDIT, AUDIT-C, and DSM-IV are globally recognized, thoroughly validated screening and assessment tools for screening alcohol problems, we decided that these 3 scales would limit our inclusion of studies that feature samples that need treatment based on a diagnostic scale.

### Data extraction, analysis, and quality assessment

Authors CM and MP (for articles dated to 2021) and CM and AL (for articles dated 2022 and 2023) independently reviewed the title and abstract of each identified article and assessed for inclusion/exclusion using Covidence (Veritas Health Innovation, Melbourne, Australia, available at www.covidence.org). In the second screening stage, full-text articles were obtained for all articles deemed by both reviewers relevant or possibly relevant (categorized as “yes” or “maybe”) based on the initial title and abstract review. Four authors/research assistants (MP, AL, CM, and EZ) independently assessed each of the full-text articles to determine their eligibility. Each article was reviewed by at least 2 team members to ensure consistency. Conflicts between the reviewers were discussed and resolved during regular screening resolution meetings with the senior author.

Authors MP and KZ (for articles dated to 2019), CM and MP (for articles dated 2020 and 2021), and CM and AL (for articles dated 2022 and 2023) independently extracted data from each of the 86 eligible articles. A data extraction spreadsheet was designed to extract information, such as the sociodemographic characteristics of the participants (e.g., sex/gender, age, race/ethnicity, socioeconomic status), study type, which alcohol screening tool(s) was/were used to assess problematic alcohol use, recruitment methods and study enrollment, characteristics of the interventions described in each study (e.g., how the intervention was delivered, by whom, where, when, modifications, and fidelity), and intervention outcomes, including attention to outcome data based on sex characteristics and/or gender identity.

### Sex and gender considerations

The language of male and female when referring to sex is often used to describe a body’s biological, anatomical, and chromosomal qualities, but where those qualities are often presumed rather than explicitly measured [26–28]. Importantly, sex development is often more complicated than the male/female binary suggests (i.e., in so far as intersex people exist, and in so far as many sex-based characteristics are more bimodal than binary) [26–28]. Further, many of these sex-based characteristics are subject to change later in life, so that a person’s sex assignment at or before birth may tell us little about their current anatomy or physiology [27]. Gender, conversely, is used to describe all of the culturally, temporally, and socially specific expectations, norms, roles, and characteristics [28]. Gender identity, specifically, refers to how someone identifies in relation to the culturally available gender identity categories, such as man, woman, nonbinary [28,29]; with further specificity involving markers of gender modality—whether someone’s current gender identity aligns with the identity they were assigned at or before birth (with sex as a proxy for the assigned gender identity). With regards to our use of language throughout, we use the term “trans” as an inclusive term, in which “trans” is a gender modality concept which refers to anyone who identifies differently than the gender they were assigned, and which captures transsexual, transgender, nonbinary, genderqueer people among others, including people who do not claim “trans” as part of their identity. Cisgender or cis is used to refer to people who currently identify with the gender they were assigned [10].

Informed by the Sex and Gender Equity Research (SAGER) guidelines [30,31], we designed our data extraction to assess the role of sex and gender in each article, for example, whether the terms sex and gender were used with precision, whether the study sample was homogenous in regards to sex and/or gender, whether sex and/or gender was a covariate in the study, whether justification was provided for the relevance of sex and/or gender as a consideration in the study, and whether the article relied on sex-based and/or gendered assessments of problematic alcohol use. Specifically, we assess sex and gender considerations within description and/or discussions regarding: eligibility criteria, participant/sample descriptions, data collection and measurement, analyses and interpretations of results, study limitations, and recommendations for future research.

### Risk of bias assessment

Considering the methodological nature of this systematic review focusing on how sex and gender are conceptualized, measured, and interpreted in a group of interventions aimed at addressing problematic alcohol use among youth, assessing the risk of bias in the included interventions was not directly relevant to our specific research question. Indeed, our review was primarily concerned with how sex and gender were accounted for in the included studies, rather than evaluating the overall quality or validity of the study findings. Therefore, the risk of bias assessment, which typically evaluates the internal validity of the individual studies, was not directly applicable to our study and interpretation of findings. To assess the quality of reporting of the interventions described in the articles, however, we used the Template for Intervention Description and Replication (TIDieR) checklist and guide [32] to extract data relating to each of the 12 items in the TIDieR checklist because it was developed to improve completeness of reporting of interventions, in an effort to improve replicability of research findings.

The extracted data from the final pool of articles was analyzed and synthesized using narrative techniques to assess how sex and gender information was collected, measured, and reported.

## Results

### Study selection

**Fig 1:**
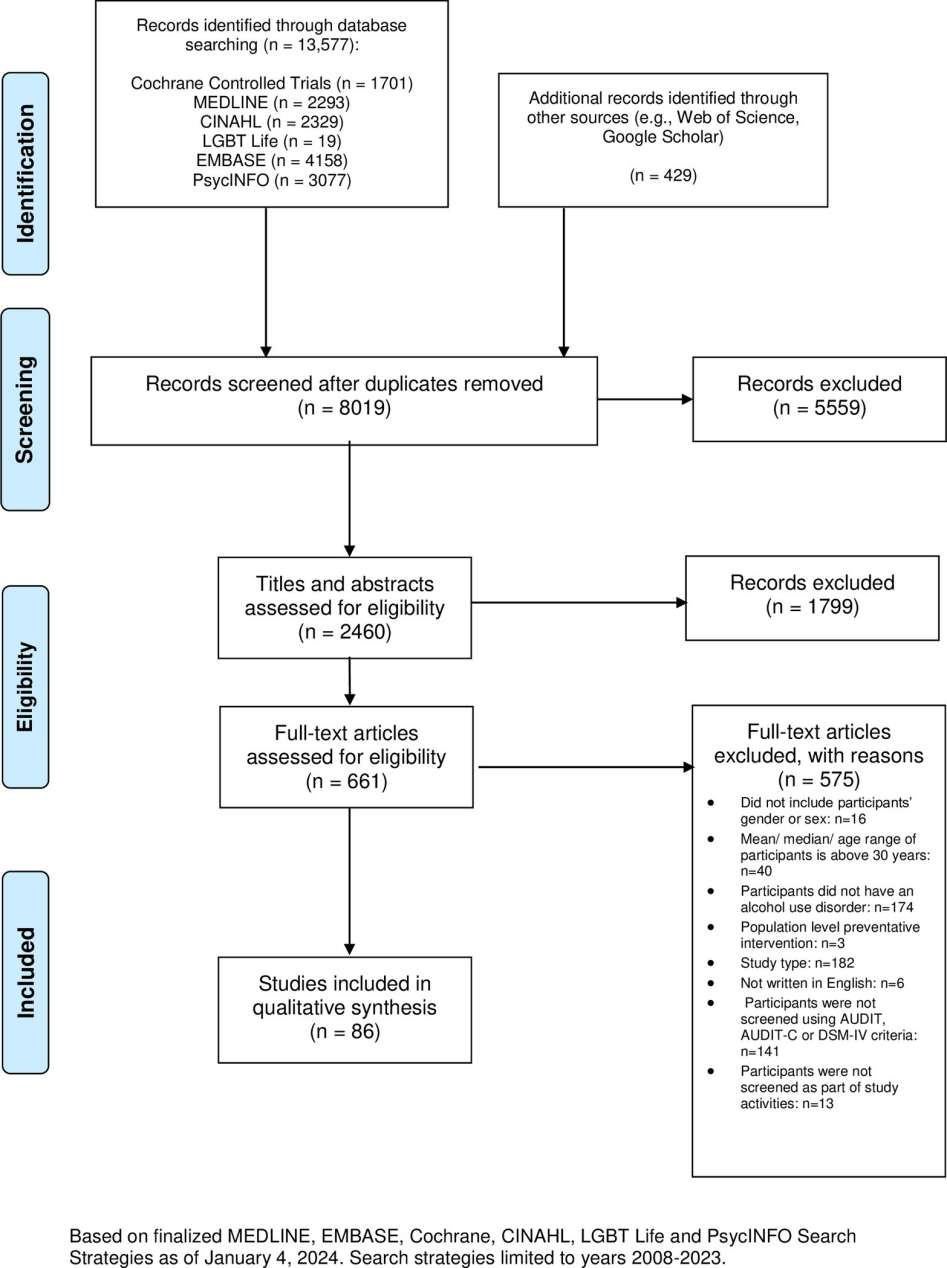
PRISMA flow diagram.

Our search strategy identified a total of 14,006 studies, of which 8,019 unique eligible records were reviewed for inclusion. Abstract and full-text screening resulted in a total of 86 studies (Fig 1).

### Study characteristics

A total of 86 articles are included in the review (Table 2). Most of the included studies used AUDIT or AUDIT-C to screen participants for alcohol use (*n* = 77). Most of the included studies are randomized controlled trials (RCTs).

**Table 2:** Study characteristics of articles included in the review.

Citation	First author, year published	Age of participants (range and/or mean and/or median)	Alcohol screening/assessment administered	Brief description of intervention	Study type
**[33]**	Andersson, 2015	Mean = 23.2	AUDIT	Brief alcohol intervention (WEB vs. Interactive Voice Response (IVR))	RCT
**[34]**	Baker, 2014	Mean = 19.36	AUDIT	Alcohol Skills Training Program (CHOICES)	Quasi-experimental
**[35]**	Bendtsen, 2015	<30 years old	AUDIT	Online alcohol intervention (AMADEUS-2)	RCT (2-arm, parallel)
**[36]**	Berman, 2019	Mean = 30.4 (for Continual Frequent-Heavy Drinkers) and 25.7 (for Iterant Frequent-Heavy Drinkers) and 29.5 (for Total Frequent-Heavy Drinkers) and 25.6 (for Total Moderate Drinkers)	AUDIT	Mobile phone brief intervention	RCT
**[37]**	Bertholet, 2015	Age 20–21 (mean = 20.75)	AUDIT	Internet-based brief intervention	RCT
**[38]**	Bertholet, 2018	Age 20.7 (mean at baseline); age 25 (mean at 47 month follow-up)	AUDIT	Internet-based brief intervention	RCT
**[39]**	Bertholet, 2023	Mean = 22.35	AUDIT	Smartphone-based alcohol intervention	RCT
**[40]**	Bewick, 2010	Age 18–67 (mean = 21.5)	AUDIT	Web-based intervention for student alcohol use	RCT (stratified, 3-arm)
**[41]**	Bogg, 2018	Age 18–23	DSM-IV	Brief educational commitment (EC) module + BASICS	RCT
**[42]**	Bold, 2016	Age 18–23 (mean = 21.4)	DSM-IV	Naltrexone vs. placebo	Double-blind placebo-controlled randomized clinical trial
**[43]**	Bonar, 2022	Mean = 20.4	AUDIT-C	Motivational interviewing	RCT
**[44]**	Büchele, 2020	Age 18–30 (mean = 20.9)	AUDIT	Brief personal feedback intervention	RCT
**[45]**	Burleson, 2012	Age 13–18 (mean = 16)	DSM-IV	Integrated motivational enhancement therapy/cognitive behavioral therapy sessions (in person vs. brief telephone vs. no aftercare)	RCT (3-arm)
**[46]**	Canale, 2015	Mean = 21.64	AUDIT	Computerized drinking motives alcohol intervention	Quasi-experimental study
**[47]**	Clarke, 2015	Mean = 23.85	AUDIT	Brief personalized feedback intervention	RCT
**[48]**	Cornelius, 2009	Age 15–20	DSM-IV	Fluoxetine	Double-blind placebo-controlled clinical trial
**[49]**	Cornelius, 2011	Mean = 19.5	DSM-IV	Cognitive Behavioural Therapy/CBT + Motivational Enhancement Therapy/MET + Fluoxetine	Acute phase trial
**[50]**	Coughlin, 2021	Age 16–24 (mean = 20.7)	AUDIT-C	Adaptive Mobile Intervention	RCT
**[51]**	Cunningham, 2012(a)	Age 14–18 (mean = 16.8)	AUDIT-C	Brief motivational interview	RCT
**[52]**	Cunningham, 2012(b)	Mean = 22.6	AUDIT-C	Web-based personalized feedback intervention (Check Your Drinking University version/CYDU)	RCT
**[53]**	Cunningham, 2015	Age 14–20 (mean = 18.6)	AUDIT	Brief alcohol intervention	RCT (2 arm)
**[54]**	D’Amico, 2018	Age 12–18 (mean = 16)	DSM-IV	Brief motivational interview	RCT
**[55]**	Davies, 2017	Age 18–30 (mean = 21.7)	AUDIT-C	Personalized digital interventions (OneTooMany vs. Drinks Meter)	RCT
**[56]**	Deluca, 2022	Mean = 16.1	AUDIT-C	Personalized feedback and brief advice	RCT
**[57]**	DiClemente, 2021	Age 18–24	AUDIT	Group Motivational Enhancement Therapy module	RCT
**[58]**	Eggleston, 2007	Mean = 19	AUDIT, DSM-IV	Brief feedback intervention	RCT
**[59]**	Frohlich, 2021	Mean = 24.6	AUDIT	Online, minimally guided, integrated program for comorbid alcohol misuse and emotional problems in young adults	RCT
**[60]**	Fucito, 2017	Mean = 20.71 for Call It A Night intervention group/mean = 20.33 for Healthy Behaviors control group	AUDIT	Integrate sleep and alcohol intervention (Call It a Night)	RCT
**[61]**	Gajecki, 2014	Mean = 24.72	AUDIT	Mobile phone brief intervention (Promillekoll vs. PartyPlanner)	RCT (3-arm, parallel, repeated-measures)
**[62]**	Gajecki, 2016	Mean = 24.7	AUDIT	Digital intervention	RCT
**[63]**	Gajecki, 2017	Mean = 25.41	AUDIT	Skill straining mobile app (TeleCoach)	RCT
**[64]**	Gaume, 2011	Mean = 19.9	AUDIT	Brief motivational intervention	RCT
**[65]**	Gaume, 2014	Mean = ~20	AUDIT	Brief motivational intervention	RCT
**[66]**	Gaume, 2021	Age = 20	AUDIT	Brief motivational interview	RCT
**[67]**	Geisner, 2015	Mean = 20.14	AUDIT	Brief web-based intervention	RCT
**[68]**	Ghosh, 2023	Age 18–21 (mean = 19.6)	AUDIT	Brief intervention, control intervention	RCT
**[69]**	Gwaltney, 2011	Age 18–24	AUDIT	Brief alcohol intervention (motivational intervention + personalized feedback vs. feedback only)	RCT
**[70]**	Heideman, 2008	Age 18–27 (mean = 20.94)	DSM-IV	Cognitive Behaviour Group Therapy/CBGT + Brief Alcohol Screening and Intervention for College Students/BASICS	RCT
**[71]**	Hides, 2018	Age 16–25 (mean = 20.4)	AUDIT	Mobile app intervention (Ray’s Night Out)	RCT
**[72]**	Hu, 2016	Age 18–23	AUDIT	Motivational interviewing + social anxiety treatment	Multiple baseline single-subject design
**[73]**	Hurlocker, 2021	Age 18–21 (mean = 19.14)	AUDIT	Motivational interview	RCT
**[74]**	Kamal, 2020	Mean = 18.97	AUDIT	Screening and brief intervention	Double-blind, parallel-group RCT
**[75]**	Kaminer, 2008	Age 13–18 (mean = 16)	DSM-IV	Integrated motivational enhancement therapy/cognitive behavioral therapy sessions (in person vs. brief telephone vs. no aftercare)	RCT (3-arm)
**[76]**	Kaminer, 2018	Age 13–18	DSM-IV	Adolescent Substance Abuse Goal Commitment	RCT
**[77]**	Karnik, 2023	Average = 22.8	AUDIT	Electronic screening and brief intervention	RCT
**[78]**	Kazemi, 2020	Study 1 mean = 19.04; Study 2 mean = 19.86	AUDIT	Brief motivational enhancement intervention	RCT
**[79]**	King, 2020	Mean = 19	AUDIT	Brief motivational enhancement intervention	RCT
**[80]**	Kypri, 2008	Age 17–24 (mean = 20.1)	AUDIT	Electronic screening and brief intervention	RCT (stratified, 4-arm)
**[81]**	Kypri, 2009	Age 17–24 (mean = 19.7)	AUDIT	Proactive Web-Based Alcohol Screening and Brief Intervention (THRIVE Study)	RCT (2-arm)
**[82]**	Kypri, 2013	Age 17–24 (mean = 20.2 for intervention group/mean = 20.1 for control group)	AUDIT-C, AUDIT	Web-Based Alcohol Screening and Brief Intervention	RCT (multi-site, double-blind, parallel)
**[83]**	Kypri, 2014	Age 17–24 (mean = 20.2 for intervention group/mean = 20.1 for control group)	AUDIT-C, AUDIT	Web-Based Alcohol Screening and Brief Intervention	RCT (multi-site, parallel, double-blind)
**[84]**	Lindgren, 2024*	Age 18–25 (mean = 20.15)	AUDIT	Narrative writing	RCT
**[85]**	Martín-Pérez, 2019	Mean = 21.01	AUDIT-C	Brief motivational interview vs. Brief cognitive behavioral therapy (bMI vs. bCBT)	RCT
**[86]**	McCambridge, 2013	Age >18	AUDIT-C	Brief online intervention	RCT (3-arm, parallel)
**[87]**	McClatchey, 2017	Age 16–19 (mean = 19.82)	AUDIT-C	Alcohol Brief Intervention	RCT
**[88]**	McGeary, 2014	Mean = 18.98	AUDIT	Alcohol-specific attention modification program	RCT
**[89]**	Miller, 2019	Mean = 19.9	AUDIT	Personalized feedback intervention	RCT
**[90]**	Oddo, 2021	Mean = 19.87	AUDIT	Brief motivational interview	RCT
**[91]**	Ostafin, 2012	Mean = 18.5	AUDIT	Brief motivational intervention	RCT
**[92]**	Palm, 2016	Age 15–22 (mean = 18.2)	AUDIT-C	Motivational interviewing	RCT
**[93]**	Paulus, 2021	Mean = 22.14	AUDIT	Personalized feedback intervention	RCT
**[94]**	Ray, 2012	Age 21–29 (mean = 22.3)	AUDIT	Naltrexone	RCT (double-blind, placebo-controlled)
**[95]**	Ridout, 2014	Age 17–24 (mean = 19.05)	AUDIT	Social norm intervention	RCT
**[96]**	Rocha, 2012	Age 18–35 (mean = 25.38)	AUDIT	Personalized feedback intervention (personalized normative feedback/PNF vs. PNF + personalized drinking feedback/PDF)	RCT
**[97]**	Shuai, 2022	Mean = 20.63	AUDIT	Functional imagery training intervention video	RCT
**[98]**	Suffoletto, 2012	Age 18–24 (mean = 21)	AUDIT-C	Text message intervention (Pittsburgh Alcohol Reduction through Text-Messaging/PART Study)	RCT (multi-site)
**[99]**	Suffoletto, 2014	Age 18–25 (mean = 22 for SA+F group/22 for SA group/21.8 for Control group)	AUDIT-C	Text message intervention (Texting to Reduce Alcohol Consumption/TRAC)	RCT (multi-site, 3-arm)
**[100]**	Suffoletto, 2015	Age 18–25 (mean = 22 for SA+F group/22 for SA group/21.8 for Control group)	AUDIT-C	Text message intervention (Texting to Reduce Alcohol Consumption/TRAC)	RCT (multi-site, 3-arm)
**[101]**	Suffoletto, 2016	Age 18–25 (mean = 22)	AUDIT-C	Text message intervention (Texting to Reduce Alcohol Consumption/TRAC)	RCT
**[102]**	Suffoletto, 2018	Age 18–25	AUDIT-C	Text message intervention (Texting to Reduce Alcohol Consumption 2/TRAC2)	RCT
**[103]**	Suffoletto, 2019	Age 18–25	AUDIT	SMS Intervention	Pilot RCT
**[104]**	Suffoletto, 2023	Mean = 22.2	AUDIT-C	Text message intervention	RCT
**[105]**	Tello, 2018	Mean = 19.84	AUDIT	Alcohol Implicit Association Test	Controlled experiment
**[106]**	Terlecki, 2010a	Mandated students: Mean = 20.12 (BASICS group) and 20.14 (control group)/Volunteer students: Mean = 20.24 (BASICS group) and 20 (control group)	AUDIT	Brief motivational intervention	RCT
**[107]**	Terlecki, 2010b	Age 18–24 (mean = 20.26 for MT group/20.29 for MC group/20.18 for VT group/19.84 for VC group)	AUDIT	Brief motivational intervention (BASICS)	RCT
**[108]**	Terlecki, 2011	Age 18–24	AUDIT	Brief motivational intervention (BASICS)	RCT
**[109]**	Terlecki, 2015	Age 18–24	AUDIT	Brief motivational intervention (BASICS)	RCT
**[110]**	Terry, 2012	Mean = 21.2 for intervention group/mean = 21 for control group	AUDIT	Screening and brief intervention	RCT
**[111]**	Tomaka, 2012	Sample/Wave 1: Age 17–38 (mean = 20.77)/Sample/Wave 2: Age 17–39 (mean = 21.53)	AUDIT	Brief motivational intervention (BASICS)	RCT
**[112]**	Tzilos, 2010	Age 18–45 (mean = 25 for intervention group/mean = 26.4 for control group)	T-ACE, AUDIT-C	Computer-based brief motivational intervention	RCT
**[113]**	Vinci, 2014	Mean = 20.13	AUDIT	Brief mindfulness intervention	Randomized experimental study
**[114]**	Walton, 2015	Age 14–20 (mean = 18.6)	AUDIT	Brief alcohol intervention	RCT (2 arm)
**[115]**	Walton, 2017	Age 14–20 (mean = 18.6)	AUDIT	Brief alcohol intervention	RCT (2 arm)
**[116]**	Weinstock, 2014	Age 18–27 (mean = 20.1 for MET group/21.0 for MET+CM group)	AUDIT	Physical activity (and motivational enhancement therapy/MET vs. MET +contingency management/CM)	RCT (2 arm)
**[117]**	Weinstock, 2016	Age 18–25 (mean = 20)	AUDIT	Physical activity (and motivational interviewing/MI + exercise contracting/EC vs. MI+ contingency management/CM)	RCT (2 arm)
**[118]**	Wolter, 2021	Mean = 24	AUDIT-C	Personalized normative feedback	RCT

*While the final publication date was 2024, this article was published online ahead of print in late 2023 and was thus included in our search strategies that included all published articles until the end of 2023.

### Sex and gender in the eligibility criteria and participant descriptions

Fifty-four (62.8%) of the 86 included studies inaccurately used sex-specific terminology to describe participants’ gender identities by stating that the participants’ genders were male and female (rather than men and women) [34–36,40,42,44–47,49,50,52–56,58,59,61–63,67,69–72,74,75,77–79,81–83,86,87,89–91,94–97,101,105,108–111,114–118]. For example, one study described how randomization was stratified by gender but inaccurately operationalized this by indicating each condition is comprised of equal proportions of “male” and “female” students [110]. Two articles (2.3%) used gender-specific terminology to describe participants’ sexes by stating that the participants’ sexes were men and women (rather than male and female) [81,83].

Twenty studies (23.3%) accurately used sex terminology to refer to their samples featuring either male or female participants and did not report on the gender identities of participants [39,41,43,48,51,60,68,73,76,80,85,93,98–100,102–104,106,107]. Conversely, 3 studies (3.5%) used the terminology related to gender identity accurately to refer to their samples featuring men and women and did not report on the sexes of the participants [33,84,113].

Among the 9 (10.5%) studies that limited enrolment to participants of only 1 sex or only 1 gender, sex- and gender-specific terminology was not defined and how these identities were assessed or measured was unclear [37,38,57,64–66,88,92,112]. Three of these studies (3.5%) stated that their eligibility criteria were limited to women, and these studies accurately deployed gender-specific terminology to describe eligible study participants [57,88,112], but it was unclear how the gender of study participants was measured. In one of these studies, the authors justified their decision to focus on one gender (women) due to the importance of alcohol interventions relating to certain reproductive capacities and experiences, including pregnancy, childbearing, and postpartum experiences [112]. As such, the authors accurately used the language of gender to refer to women participants in their study; however, in limiting their eligibility only to women, they nevertheless conflated sex and gender, since pregnancy, childbearing, and postpartum are experiences limited to female sex-assignment, where people who do not identify as women can and do get pregnant. The accurate use of gender/sex concepts expands beyond how participants are themselves described and is also an important part of determining eligibility criteria. Prospective participants may be inadvertently excluded from a study, despite being eligible, if gender identity is used instead of a more appropriate shared characteristic for sex-based rationale, specifically with regards to reproduction, including pregnancy, childbearing, and the postpartum experiences [30]. Six (7.0%) of the studies which limited their study populations to a single sex were limited to male participants; 5 included specifically young Swiss males who were subjected to a mandatory army recruitment process for all male citizens beginning at the age of 19 [37,38,64–66]. These participants were described in the article as both male and men interchangeably, without indicating as to whether the participants were asked about sex and/or gender. One of the studies (1.2%) which only included male participants did not explain or justify why they decided to do so [88].

**Fig 2:**
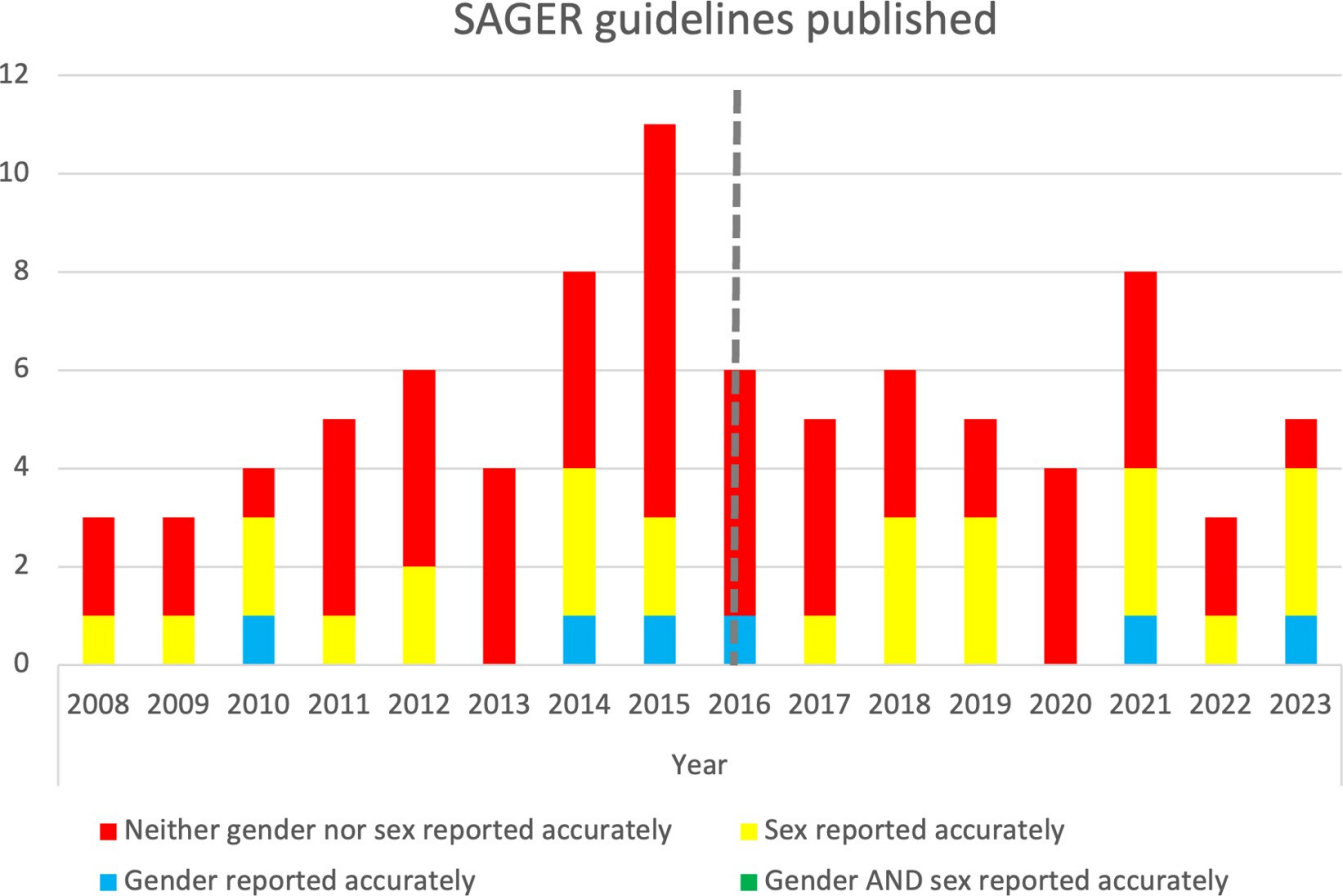
Number of articles across time that accurately reported: sex; gender; both sex and gender; or neither sex nor gender.

Therefore, from the 16-year timespan of 2008 to 2023, among the 23 articles that reported either sex or gender accurately (but not both, since none of the articles included reported both accurately), in addition to the 9 articles that limited enrolment to participants of only 1 sex or only 1 gender and did so accurately (though with some problems as described above), 15 of 44 (34.1%) were published in the first 8 years of 2015 and earlier and 17 of 42 (38.6%) were published in the next 8 years of 2016 to 2023. While this represents an increase of approximately 4.5%, the difference is not significant (*P* = 0.54); therefore, we did not observe improvements in how sex or gender were reported over time (Fig 2).

Overall, none of the 86 studies included intersex people and none acknowledged the limitations of binary sex assignment for the purposes of interpretation. That is, none of the studies stated explicitly whether their male/female participants were intersex or endosex (that is, not intersex). The language of male/female was used without further qualification. Further, none detailed how male/female may be insufficient for determining the current anatomy or physiology of participants. Further, only 2 studies reported the inclusion of trans participants [84,110]. By characterizing the sample as featuring male, female, and transgender participants, the first study conflated gender modality (transgender) with sex (male, female) [110]. The second characterized the sample as featuring women, men, and “gender-diverse” participants at some points in the article and women, men and “other gender identity” at others [84], thereby conflating gender identity and gender modality. Given the authors did not report on gender modality (trans and cis), we are unable to assess if trans people are also included in the categories of men and women. Further, neither study differentiated between trans people of different genders in their respective “transgender” and “gender-diverse/other gender identity” categories; these participants were instead aggregated together into a single category, likely comprised of trans men, trans women, nonbinary people, and others.

### Collection and measurement

We assessed the 86 articles for how they collected and measured the sexes and/or gender identities of study participants and how they described the measurement process. Eighteen (20.9%) of the studies used self-reporting instruments to collect the sexes of their participants and the articles neither specified whether the participants were offered options beyond male or female, nor whether participants were asked whether they had a variation in their sex development/were intersex. The articles also did not clearly indicate whether participants were asked specifically for their at-birth sex assignments (for example, as compared to their legal sexes, which may be different) [50,51,59,73,74,78,81,83,85,90,97–100,102,103,106,107]. Ten of these 18 studies were found in the previous section above to have accurately used sex terminology [51,73,85,98–100,102,103,106,107]; however, information about the self-reporting measures were not provided so it is not possible to determine whether sex was accurately measured since the sex terms male and female can be used to describe both at-birth assignment and legal sex, and since neither sex assignment nor legal sex is sufficient to ascertain anatomy or physiology. Eight of the 18 studies [50,59,74,78,81,83,90,97] were among those that conflated sex and gender terminology and, as such, we are unable to determine precisely what was measured—sex assignment, legal sex, gender identity, or some other variable.

Twenty-nine studies (33.7%) used self-reporting instruments to collect the gender identities of their participants. Similarly to reporting “sex,” articles did not specify whether gender identity was self-reported using open-text or, if researcher-provided response options were used, which identities were provided [33,34,36,40,42,46,49,53,58,61,67,72,82,87,89,91,94–96,101,105,109–111,113–117]. Only 2 of these 29 studies were previously determined to have accurately mobilized gender identity-related terminology [33,113]. The remaining 27 articles were among those that conflated sex and gender terminology [34,36,40,42,46,49,53,58,61,67,72,82,87,89,91,94–96,101,105,109–111,114–117]. It is therefore unclear what was ultimately measured and how. For example, it is unclear whether participants were offered only the binary gender identity options of man and woman. Based on the pervasive conflation of gender and sex in these studies, it is possible that some studies asked participants for their gender identities, but offered sex terms (e.g., male or female) as response options, despite using gender terms to later describe their samples.

Thirteen (15.1%) of the studies reported that they collected the sexes of participants [39,41,43,44,48,60,68,76,79,80,93,104,118]. A further 14 (16.3%) studies reported collecting the genders of participants [45,47,52,54–56,62,63,70,71,75,77,84,86]. However, none of these 27 studies (31.4%) described how they undertook the task of assessing, collecting, and measuring the sexes and/or genders of the participants. Among the 9 studies (10.5%) that included participants of only one sex or gender, no details are provided about how the sexes or genders of those participants were collected and measured [37,38,57,64,65,66,88,92,112]. Without describing their data collection methods and measures, it was unclear how these 36 studies collected and measured participants’ gender identities and/or sexes, making it difficult to assess whether these were used precisely, accurately, and inclusively in their approaches to data collection and measurement.

### Sex and gender in the analyses and interpretations of results

In 54 of the studies (63.0%), sex and/or gender was used as a covariate in the analysis, and this was often done to control for the effects of sex and/or gender on their intervention [33–35,39–51,53–56,58–61,63,70,71,75–78,80–84,86,89,90,93,96,99–103,105,106,109–111,114,117,118]. For example, Tello and colleagues [105] considered gender as a potential factor in the a priori power analysis but ultimately found that it did not impact their dependent variable and therefore excluded it from their subsequent analyses. However, they noted that their participants were mostly female (which is a sex term) and that although they “did not find any effect of gender on [the] results, further research is needed to test whether evaluation conditioning is equally efficient across gender” [105]. Given the conflation of sex and gender terms throughout the article, it is not possible to determine whether the authors ultimately controlled for sex or for gender, as well as whether they were suggesting that more testing is needed across gender-related factors, sex-related factors, or both.

In 64 of the studies (74.4%), the authors did not discuss whether sex or gender were relevant to their hypothesis or analysis [33,35–41,43–56,58–60,62,66–89,93,95–97,106–109,111,113–116,118]. Half of the 86 studies in the sample (*n* = 43, 50%) did not mention sex or gender in their discussion sections, neither as variables which were significant or relevant to their findings, nor as factors that they explicitly featured in their recommendations for practice or policy based on their findings [33,35,39,41,47–49,51–56,58–60,66–70,73,77–78,80,82–84,86,88,90,91,93,94,99–104,110,114,116]. Although the authors conflated sex and gender terms throughout their article, Bewick and colleagues addressed how sex/gender affected study results by stratifying the results by sex, discussing how the regression analysis “showed that males entered the study with a higher total number of units consumed over the last week,” and how these findings are in agreement with other literature [40].

### Sex, gender, and study strengths and limitations

Twelve studies (14.0%) identified the relative homogeneity of their sample (i.e., samples that were composed of entirely or mostly 1 sex or gender) as a limitation of their research [37,38,44–46,64,76,87,93,97,105,113]. For example, Canale and colleagues [46] described how their sample being comprised predominantly of female participants was a study limitation and argued that future research ought to better integrate and consider sex and/or gender variables. Five studies (5.8%) described gender-related limitations in terms of the generalizability of their findings [34,50,85,89,96]. For example, Miller and colleagues [89] reported that having a higher proportion of women in the control than the intervention group was a limitation.

Seven studies (8.1%) both discussed sex and/or gender within the context of their findings and recommended that sex and gender ought to be more fulsomely integrated into future research in the area of youth alcohol interventions [40,42,57,61,62,65,106]. A further 3 studies (3.5%) recommended that sex and gender ought to be integrated into future research, though they did not discuss sex and/or gender within the context of their own findings [43,107,115]. One study recommended that future research ought to explicitly explore intervention outcomes among sexual and gender minority populations [43]. One study (1.2%) discussed sex and/or gender only insofar as they provided citations from previous research studies but did not discuss sex and/or gender in the context of their current findings [112]. Thirteen studies (15.1%) discussed sex and/or gender in relation to their current findings but did not expand on how sex and/or gender impact alcohol interventions and other broader implications [36,63,71,72,75,81,92,98,108,109,111,117,118]. For example, Gajecki and colleagues [63] discuss the gender differences in participant outcomes in the study (e.g., “Analyses by gender showed that men in the intervention group compared to men in the assessment-only control group had higher odds ratios for not having excessive alcohol consumption than women in the intervention group compared to women controls”) but did not expand on the implications of these findings. Finally, concerns over generalizability were limited to how or whether the findings could be generalized to all men and women or males and females. None of the studies discussed the relevance or generalizability of the findings for intersex and trans people.

#### Quality of reporting of intervention characteristics

Overall, the quality of reporting about interventions in the included articles was good. Only 4 articles addressed every item on the TIDieR checklist with all relevant details [39,56,58,84], but most articles (*n* = 48, 56%) included all relevant details for 8 of the 12 items in the TIDieR checklist ([32]; Table 3).

**Table 3:** TIDieR Checklist Item scores for articles included in the review.

Checklist item	Item description	Number and percentage of articles which addressed all elements of this item	Number and percentage of articles which addressed some elements of this item	Number and percentage of articles which did not address this item	Number and percentage of articles for which it was unclear whether they addressed this item
**Item 1: Brief name of intervention**	Brief name of intervention: Is the name precise, well described, and is it easy to identify the type of intervention based on the name?	83 (96.5%)	3 (3.5%)	0	0
**Item 2: Why**	Do the authors describe the rationale and theory of goal of the elements essential to the intervention?	73 (84.8%)	13 (15.1%)	0	0
**Item 3: What (materials)**	Do the authors provide a full description of the physical or information materials used in the intervention?	49 (56.9%)	22 (25.6%)	15 (17.4%)	0
**Item 4: What (procedures)**	Do the authors describe each of the procedures, activities, and/or processes used in the intervention, including any enabling or support activities?	73 (84.9%)	13 (15.1%)	0	0
**Item 5: Who provided the intervention**	Do the authors describe the intervention provider(s), how many there were, their role, their job title, and their expertise and skills?	47 (54.7%)	25 (29.1%)	14 (16.3%)	0
**Item 6: How (mode of delivery)**	Do the authors describe how the intervention was delivered (individually/group; face-to-face/virtually)?	86 (100%)	0	0	0
**Item 7: Where (types of locations, infrastructure)**	Do the authors describe the location where the intervention is delivered? Do these descriptions include relevant details?	62 (72.1%)	13 (15.1%)	5 (5.8%)	6 (7.0%)
**Items 8: When and how much**	Did the authors describe the number of times the intervention was delivered and over what period of time including the number of sessions, their schedule, and their duration, intensity, or dose?	61 (70.9%)	23 (26.7%)	1 (1.2%)	1 (1.2%)
**Item 9: Tailoring**	If the intervention was planned to be personalized, titrated, or adapted, did the authors describe what, why, when, and how?	51 (59.3%)	12 (14.0%)	21 (24.4%)	2 (2.3%)
**Item 10: Modifications**	Did the authors describe any changes that occurred during the course of the study?	12 (14.0%)	1 (1.2%)	73 (84.9%)	0
**Item 11: How well (planned)**	If the intervention adherence of fidelity was assessed, did the authors describe how and by whom. If any strategies were used to maintain or improve fidelity, did they describe them?	35 (40.7%)	11 (12.8%)	39 (45.3%)	1 (1.2%)
**Item 12: How well (actual)**	If intervention adherence or fidelity was assessed, did the authors describe the extent to which the intervention was delivered as planned?	45 (52.3%)	24 (27.9%)	17 (19.8%)	0

## Discussion

Our findings identify how the vast majority of alcohol treatment intervention research with youth are conflating sex and gender factors, including terminologically, conceptually, and methodologically. None of the 86 studies defined, measured, and reported both sex and gender variables accurately and consistently. Most of the studies reviewed used gender and/or sex as a covariate to control for the effect of sex and/or gender on the intervention. Many studies identified limitations regarding sex and/or gender, including sample homogeneity, generalizability of findings, and the need for more research. Only 2 of 86 articles acknowledged the presence of trans people, albeit in ways that conflated gender modality with sex or gender identity. The incorrect conflation of sex and gender terms occurred across the studies and persisted over time (from 2008 to 2023), and only a small subset (*n* = 32) of the studies defined, measured, and reported either sex or gender identity accurately. None of the studies described how they assessed participants’ sexes, gender identities, or modalities (e.g., the measures they used), though just over half of the studies indicated that this was done using self-reporting instrument tools. Despite these shortcomings, the overall quality of reporting about interventions as assessed by the TIDieR checklist was good.

The omission and exclusion of trans people in research is a long-standing issue and is particularly dangerous when trans people have elevated risk for harms, as is the case for substance use [119] and alcohol use [8,120]. In the absence of a clear integration of sex and gender terms and measures, we worry that a lack of rigour in this area could result in the systematic assignment of sex and/or gender variables to participants and samples based on crude proxies, assumptions, or guesses about participants’ sexes and/or genders. For example, despite referring to the participant sample as being comprised of a certain number of men and women, and accurately calling this classification “gender,” there is a real possibility that the authors were assuming that the participants were men and women based on presumptions about the participants’ gender expressions, sexed bodies or based on other factors, including when this is done in cisnormative ways (e.g., where a person with breasts, who is wearing a dress, is assumed to be a woman, despite their identifying as nonbinary). At this juncture, it is clear that the body of youth alcohol intervention research widely relies on data collection and reporting approaches that presume (and therefore replicate) sex and gender binaries, thereby resulting in the systematic exclusion of intersex and trans people.

While long-standing confusions and conflations of terminology in the sex and gender field are well documented [1,2,121], we are also concerned that the lack of precision and analytic rigour is inhibiting progress with regards to youth alcohol treatment interventions capacity to account for sex- and gender-related factors. For example, we found that most of the study designs seem to be based on a “sex differences” paradigm, an approach in which sex measures are collected to examine the differences between bodies that were assigned male or female [121]. However, at the level of analysis, sex differences tended to be used almost exclusively for descriptive rationale and discussed and interpreted only in ways that treated these differences as separate, dichotomous, and non-overlapping [1,2]. Similarly, for the subset of studies that would ostensibly fall within a “gender differences” paradigm—an approach that seeks to understand the social and cultural experiences within and across genders [121]—gender differences were also used almost exclusively for describing the sample and not considered within the analysis or interpretation of results. Given that the use of both sex and gender paradigms are largely used primarily to describe (accurately and inaccurately) study samples, it remains unlikely that this approach to sex and gender science will have the capacity to advance interventions that fulsomely account for or address sex- and gender-related factors.

None of the studies included in our review were designed in a way that they could identify both sex- and gender-related factors (i.e., the components, factors, and/or processes associated with sex and those associated with gender) impacting alcohol- and intervention-related outcomes. For example, given that rudimentary and foundational understandings of sex and gender factors were absent, it is perhaps unsurprising that none of the studies assessed or considered sex and gender interactions (experiences of having a sexed body in a gendered social context) and the real-world experiences and impacts of these interactions on alcohol treatment intervention outcomes for intersex and endosex, cis and trans youth of all genders [1,2]. We do not necessarily consider this as a problem, as it may be the case that either sex- or gender-related factors are irrelevant to a given research question or intervention (e.g., behavioral interventions where sex factors like anatomy and physiology are not part of the mechanistic processes) and it is therefore reasonable to only include one or the other. Indeed, it is important from both ethical and methodological perspectives that researchers define, measure, and report only those measures that are relevant to their research questions and mechanistic hypotheses, rather than reifying the importance of variables like sex in research where sex does not feature in the mechanistic hypothesis. Further, where sex is deemed relevant to a given study or intervention, it is imperative that researchers identify the sex-specific factors that impact outcomes (e.g., hormones), recognizing that male and female (as assignments or legal categories) are not appropriate proxies for these more specific and precise factors (e.g., where there are people assigned male with low testosterone and people assigned female with high testosterone, which could only be ascertained by measuring not sex, but hormone levels). Still, there are research questions and interventions that should include measurements of both sex and gender (identity and modality), including, for example, pharmacological interventions that seek to assess the impacts of human physiology, anatomy, hormones, enzymes, genetics, and neurobiology (sex-related factors) when combined with behavioral or structural interventions that may feature impacts or outcomes associated with gender roles and norms, gender relations, gender identities, gender modalities, and institutionalized gender.

In addition to issues with the terminology (which impacts not only how participants are described, but inclusion/exclusion criteria, and which is an important part of data collection and measurement), the studies in our review also relied on validated tools for assessing problematic alcohol use which themselves likely contributed to the misuse and exclusionary approach to gender and sex in the scientific research described in this review. Although we do not hold the authors of the 77 studies who used AUDIT and AUDIT-C accountable for the sex- and gender-based limitations of these tools, we note that none of these studies did so in a way which indicated an awareness of the cisnormative assumptions embedded within these tools and the resulting shortcomings to their applicability [122,123]. We note that it is also likely that the cisnormative conflation of sex and gender at the level of these alcohol screening tools contributes to “knock-on” effects in other areas of the research design in which sex and gender are deployed inaccurately. We argue that if the screening tools substantiated their use of different thresholds for different kinds of people through a more careful articulation of sex and/or gender concepts, those working in this area (including clinicians who use these tools in their clinical practice) would be compelled to consider sex and gender constructs more precisely and accurately [123]. At this juncture, we follow Flentje and colleagues and the Canadian Centre on Substance Use and Addiction’s conclusions regarding the need for gender-inclusive AUDIT scores [5,122,123].

Until these issues are more fulsomely addressed in alcohol treatment intervention science, inclusivity considerations are likely to remain unaddressed in this area. For example, while an emerging evidence base reveals that trans people have higher rates of alcohol use as compared to their cisgender counterparts [7], the alcohol treatment intervention research does not account for trans youth. Based on our review, it appears that sex and gender minority populations are being systematically excluded from research, thereby resulting in imprecise or non-inclusive recommendations for these populations in the design and implementation of treatment interventions. Intersex and trans people have well-established and justifiable mistrust with academics, researchers, and clinicians alike, an issue that is likely exacerbated by study protocols and tools that do not provide opportunities for meaningful inclusion [124–129]. Arguably, even if intersex and trans people had been recruited to the studies we reviewed, it remains unclear as to whether the studies would have had the tools to meaningfully, accurately, and inclusively measure their specific sex- and gender-based factors in the study protocols and whether the researchers would have had the skill and expertise to meaningfully analyze the resulting data.

To our knowledge, our review is the first to consider over a decade of research on how gender and/or sex are mobilized in alcohol interventions for youth. We are unaware of any other review investigating the mobilization of sex and/or gender in substance use interventions that has screened over 8,000 articles. Furthermore, this review also used rigorous methods to ascertain to what extent sex and/or gender were incorporated in all facets of research design, including eligibility criteria, sample descriptions, data collection processes, analyzes, results and discussion sections, implementation considerations as well as study-specific strengths and limitations. Because of this careful screening, we were able to identify with confidence that alcohol intervention research with youth is consistently misusing sex and gender terminology in the reporting of various stages of the research cycle [8]. Another strength of this paper is that the quality of reporting of the included articles was assessed using the TIDieR checklist [32].

In terms of limitations, where sex and/or gender sociodemographic variables are considered, we would argue that these can never be divorced from race, age, class, or disability. Our systematic review does not undertake an intersectional analysis of these variables, and instead looks at how the 86 studies mobilize sex and/or gender variables in isolation rather than in an intersectional way. Furthermore, there is currently no scale to quantify or qualify the degree to which dominant norms such as endosexnormativity (the presumption that humans are naturally sexually dimorphic and where endosex lives are anticipated and valued) and cisnormativity (the presumption that binary sex and binary gender will align in predictable ways, and where cisgender lives are anticipated and valued) featured within various facets of the studies.

Finally, while our review provides answers to some of our narrowly defined review questions, we did not assess how these variables were mobilized in the interventions themselves, including whether there were sexed and/or gendered impacts of the interventions. However, had we done so, based on our findings, we anticipate that these intervention-specific results were likely also written in ways that conflated and confused these variables. For example, we do not consider whether and how interventions were tailored based on sex- and/or gender-related factors and subsequently whether the study results vis-à-vis the intervention are therefore reliable based on how that tailoring was undertaken.

To advance sex and gender science in this domain, our findings underscore the importance of including checklists for reporting on sex and gender in medical research as a necessary requirement by funders and peer-reviewed journals (e.g., the SAGER checklist [31]). By implementing these requirements and adopting improved reporting practices, authors would be compelled to consider and address, where relevant, sex and gender factors in their interventions. This would not only enhance the quality and relevance of interventions in addressing harmful alcohol use among youth but also ensure that these interventions take into account the nuanced influences of sex and gender on individual responses to different alcohol treatment approaches. As a result, more accurate findings can be obtained, leading to better-informed decision-making and improved health outcomes among youth who use alcohol. Nevertheless, challenges remain with how to advance guidelines such as SAGER across the health sciences ecosystem; indeed, the current review observed no statistically significant improvements with regards to how sex and/or gender are reported pre- and post- the 2016 publication of the SAGER guidelines, likely due to resource limitations at journals, concerns about mandating changes, and lack of awareness or resistance [130]. We agree with others that improving research and reporting practices will require wider involvement of pertinent parties from across journals, universities, professional societies, ethics committees, funders, industry, and policy makers [130].

To move the science forward in this area, it will also be important that researchers clearly articulate whether the mechanistic hypotheses are related to sex, gender, or both, and to advance study designs and procedures that can accurately, precisely, and inclusively account for sex and gender. As we have argued elsewhere, intervention research should also be designed to assess differential effects, including by gender. To measure gender differences, steps such as conducting stratified analyses, testing for interaction effects, and performing subgroup analysis should be followed. Indeed, even in an RCT with balanced groups of (cis and trans) men and women, gender differences in response to the intervention can still exist [131]. Stratifying the analysis by gender will provide statistical insights into subgroup differences, assessments of clinical relevance, and allow for further exploratory analyses based on gender differences. Ultimately, stratifying the analysis by gender—including in ways that are attentive to nonbinary gender identities—and employing appropriate statistical methods will help identify meaningful differences in treatment responses between and across different genders.

There is also a need to include intersex and trans people in study designs that accurately describe study samples using the appropriate and corresponding sex and/or gender language. Descriptions and discussions of the limits to generalizability are needed if subgroups are excluded, including if intersex and trans people are underrepresented. Drawing on the sex and gender science methods literature about best practices for measuring gender modality (e.g., the commonly used two-step method [132]) and providing corresponding details about the approach used for measuring participants’ sexes, gender identities, and/or gender modalities, including by listing measures and response options in text or via supplemental data files, will be important to moving this field forward. Finally, ensuring that sex and/or gender data are analyzed, interpreted, and discussed in ways that attend to the complex sexed and/or gendered factors which impact the lives and alcohol-related experiences of study participants will be critical in our efforts to advance youth alcohol treatment interventions.

In summary, the significant methodological problems identified in our review expose an evidence base that lacks the capacity to inform sex- or gender-based approaches to alcohol treatment intervention responses for youth. Moving forward, it will be imperative for researchers to deploy sex and gender as unique and specific variables with appropriate terminology available to measure, describe, and assess the implications, where precision in understanding and interpreting these constructs will improve the overall quality of the evidence base to address alcohol-related harms. It is also imperative that sex and gender variables are used in a way that ensures that intersex and trans people are meaningfully integrated so both research and intervention can address their alcohol-related needs [133–138].

## Supporting information

S1 PRISMA Checklist PRISMA 2020 checklist. (DOCX)

S1 Appendices Appendix A. Amendments to information provided at PROSPERO registration. Appendix B. Medline search strategy.
(DOCX)

## Abbreviations

IPV: intimate partner violence
RCT: randomized controlled trial
SAGER: Sex and Gender Equity Research
TIDieR: Template for Intervention Description and Replication
