# Supplementary material for: A sex- and gender-based analysis of alcohol treatment intervention research involving youth: A methodological systematic review
Source: PLoS Med. 2024 Jun 3;21(6):e1004413. doi: 10.1371/journal.pmed.1004413 (PMC11182506; doi:10.1371/journal.pmed.1004413)
Supplement: S1 Appendices — Appendix A. Amendments to information provided at PROSPERO registration. Appendix B. Medline search strategy. (DOCX) [file pmed.1004413.s002.docx]

**Appendix A**

**Amendments to information provided at PROSPERO registration**

**11. Team members and organizational affiliations:**

In addition to the team members listed in the protocol, we also worked with the following people on the review:

- Kimia Ziafat, British Columbia Centre on Substance Use
- Ehsan Moazen Zadeh, British Columbia Centre on Substance Use
- Julien Quesne, Université de Montréal

They are listed in the manuscript as an author (KZ), or in the acknowledgement section (EZ and JQ).

**15. The review question** **evolved** from “How are gender and sex measured and reported in research on alcohol treatment for youth (ages 13-24)?” (protocol) to “How are gender and sex measured and reported in research on alcohol treatment for youth up to age 30?” (manuscript).

We decided to expand our definition of youth to less than 30 years old based on prior experience with youth-focused research, the state of public health research regarding youth and alcohol use, and trends among individuals within this age range, including delayed transitions into adulthood. More details in the manuscript under the heading “Data extraction, analysis, and quality assessment.”

**19. The summary criteria for participants studied by the review changed.**

Protocol: We will include studies with participants who: (a) have their gender and/or sex recorded in the data (b) are between the ages of 13-24 at the time of data collection (c) use alcohol to a degree that has been defined as “problematic” by the original study’s definition (d) participated in alcohol treatment and care intervention.

Manuscript: We included studies where (a) participants had their sexed and/or genders gathered and recorded in the data, (b) participants were less than 30 years old at the time of data collection, (c) participants were screened using AUDIT, AUDIT-C or a structured interviewing using DSM-IV criteria for problematic alcohol use, and screening occurred as part of the study activities.

Changes to the age of included participants are discussed for item 15 above. These changes were made in order to clarify the scope of the review and to provide a clearer parameterization of harmful alcohol use.

**26. Data coders:** A.J. Lowik, Kimia Ziafat, Ehsan Moazen Zadeh and Julien Quesne were also involved in the selection and coding of data.

**27. Risk of bias was not assessed** for this review because it was not directly relevant to our specific research question.

We decided to use the TIDieR checklist, and forgo using GRADE, because we were interested in evaluating the reporting of interventions in the studies included in the review.

**36. We changed the keywords** associated with the paper for better accessibility and findability.

39. We updated the search two times prior to publication to include papers from 2020-2021 and 2022-2023. The same search strategy was used each time.

## Appendix B

**Medline Search Strategy**

Database: Ovid MEDLINE(R) and Epub Ahead of Print, In-Process & Other Non-Indexed Citations and Daily <1946 to December 31, 2023>

Search Strategy:

--------------------------------------------------------------------------------

1 youth*.ti,ab.

2 (young adj2 (adult* or people or person* or male* or female* or m?n or wom?n or transgender* or transsexual*)).ti,ab.

3 (adolescen* or teen*).ti,ab.

4 ((emerging or early) adj2 adult*).ti,ab.

5 (millennial* or juvenile* or minor* or pupil* or student* or adolescen* or girl* or boy* or youth* or child or children or teen* or minors or juvenil* or pubert* or pubescen* or pre-pube* or kid or kids or underage*).ti,ab.

6 ((high?school* or secondary school* or college* or universit* or post?secondary school*) adj1 student*).ti,ab.

7 1 or 2 or 3 or 4 or 5 or 6

8 exp Alcohol-Related Disorders/

9 alcohol abstinence/ or exp alcohol drinking/

10 Alcoholics/

11 alcoholic*.ti,ab.

12 alcoholism*.ti,ab.

13 ((risk* or problem* or excess* or heavy or heavily or hazard* or binge or harm*) adj4 (alcohol* or drink*)).ti,ab.

14 ((alcohol* or beer* or wine* or spirit* or liquor*) adj3 (drink* or heav* or consum* or intoxicat* or abus* or misus* or use* or excess* or addict* or depend* or beverag* or disorder* or problem* or bing* or risk* or reckless*)).ti,ab.

15 (alcohol* and (drunk* or incident* or safety or offen* or abus* or disorder* or harm* or violen* or injur* or intoxicat* or assault*)).ti,ab.

16 ((drunk* or drink*) and driv*).ti,ab.

17 (drink* adj3 (alcohol* or heav* or consum* or intoxicat* or abus* or misus* or use* or excess* or addict* or depend* or beverag* or disorder* or problem* or bing* or risk* or reckless*)).ti,ab. (39153)

18 drunk*.ti,ab.

19 (binge* adj1 drink*).ti,ab.

20 Underage Drinking/

21 8 or 9 or 10 or 11 or 12 or 13 or 14 or 15 or 16 or 17 or 18 or 19 or 20

22 7 and 21

23 exp Alcohol Deterrents/

24 alcohol reduction*.ti,ab.

25 alcohol intervention.ti,ab.

26 (rehab* or detox* or pharmacotherapy).ti,ab.

27 (Acamprosate* or Campral* or Anticonvulsant* or Antidepressant* or Baclofen* or Lioresal* or Benzodiazepine* or Buspirone or Diazepam* or Disulfiram* or Antabuse* or Gamma-hydroxybutyrate* or Naltrexone* or Revia* or Vivitrol* or Topiramate* or Topamax* or Olanzapine or Quetiapine or Aripiprazole or Memantine or Pregabalin or Gabapentin or Sertraline or Ondansetron).ti,ab.

28 pharmacological intervention*.ti,ab.

29 23 or 24 or 25 or 26 or 27 or 28

30 22 and 29

31 Mass Screening/

32 Interview, Psychological/

33 ((interview* or encounter* or visit*) adj3 (counsellor* or counselor* or nurse* or physician* or doctor* or clinician* or psychologi* or social worker*)).ti,ab.

34 psychotherapy/ or psychotherapy, brief/ or Psychotherapy, Multiple/

35 family therapy/

36 (psycho-therap* or psychotherap* or family therapy).ti,ab.

37 ((psycho-therap* or psychotherap* or counsel* or solution-focus?ed or interven* or prevent* or advice or advis* or session) adj5 (brief* or short* or short-rang* or short-term or abbreviate* or concise or early or minimal or limited or time-limited or crisis or crises or emergency or emergencies or urgent or immediate* or minimal* or minimum or quick*)).ti,ab.

38 (cognitive restructuring or Cognitive reframing or Behavio$ activation or Activity Scheduling or Problem Solving or Psychodynamic).mp. or Interpersonal Psychotherapy.ti,ab.

39 Cognitive Therapy/

40 (Selfhelp or Self Help or Self-help).ti,ab.

41 (Selfmanag$ or Self manag$).ti,ab.

42 (Selfadminister$ or Self administer$).ti,ab.

43 (((((social skill or stress management training or supportive expressive therapy or case management or Relaxation Therapy or social support or self-control training or coping skill or neurobehavioral* or brief motivation* intervention* or cognitive) adj therap*) or cognitive behavioural coping skill* training or cognitive?behavioural therapy or behavio?r) adj therap*) or alcoholic*adj2 anonymou*).ti,ab.

44 ((cogniti* or behavior* or behaviour* or conditioning or motivation* or psychosocial* or psycho-social* or psychological) adj3 (therapy or therapies or therapeutic or interven* or modify or modifies or modified or modification)).ti,ab.

45 counseling/ or directive counseling/ or motivational interviewing/

46 (counselling or counseling).ti,ab.

47 "referral and consultation"/ or physician self-referral/ or secondary care/ or tertiary healthcare/

48 (refer or refer?ing or refer?ed or refers or referral*).ti,ab.

49 (alcohol screening or brief intervention* or brief prevention* or brief alcohol intervention or therapeutic communities or vocational rehabilitation).ti,ab.

50 ("early intervention" or alcoholic*adj2 anonymou*).ti,ab.

51 ((cogniti* or behavior* or behaviour* or motivat* or psychosocial* or psycho-social* or psychological) adj3 (therapy or therapies or therapeutic or interven* or interview* or session* or modify or modifies or modified or modification or chang* or conditioning)).ti,ab.

52 (contingency adj management).ti,ab.

53 Distance Counseling/ or pastoral care/

54 Motivation/

55 motivational enhancement therapy.ti,ab.

56 ((motivation* or motivational) adj interview*).ti,ab.

57 exp Psychotherapy/

58 prize-based contingency management.ti,ab.

59 "Reinforcement (psychology)"/

60 Rehabilitation/

61 Self-help Groups/ or alcoholics anonymous/

62 (self adj2 help adj2 group*).ti,ab.

63 Voucher*.ti,ab.

64 ((twelve adj2 step) or (alcoholism* adj2 step)).ti,ab.

65 Psychiatric Rehabilitation/

66 Behavior Therapy/

67 31 or 32 or 33 or 34 or 35 or 36 or 37 or 38 or 39 or 40 or 41 or 42 or 43 or 44 or 45 or 46 or 47 or 48 or 49 or 50 or 51 or 52 or 53 or 54 or 55 or 56 or 57 or 58 or 59 or 60 or 61 or 62 or 63 or 64 or 65 or 66

68 29 or 67

69 22 and 68

70 (randomized controlled trial or controlled clinical trial).pt. or randomized.mp. or randomised.mp. or groups.tw. or placebo.mp. or clinical trials as topic.sh. or clinical trials.tw. or trial.tw. or trials.tw. or double blind.tw. or single blind.tw. or experiment*.tw. or (pretest or pre test).tw. or (posttest or post test).tw. or (pre post or prepost).tw. or before after.tw. or (quasi randomised or quasi randomized or quazi randomised or quazi randomized).tw. or stepped wedge.tw. or preference trial.tw. or natural experiment.tw. or ((quasi or quazi) adj experiment*).tw. or (staggered enrolment trial* or staggered enrollment trial*).tw.

71 (non randomized or non randomised or nonrandomized or nonrandomised).ti,ab. (21853)

72 70 or 71

73 69 and 72

74 limit 73 to humans

75 limit 74 to yr="2008 -2021"

***************************
